# The influence of inter-hospital transfers on mortality in severely injured patients

**DOI:** 10.1007/s00068-022-02087-7

**Published:** 2022-09-01

**Authors:** Job F. Waalwijk, Robin D. Lokerman, Rogier van der Sluijs, Audrey A. A. Fiddelers, Dennis den Hartog, Luke P. H. Leenen, Martijn Poeze, Mark van Heijl, on behalf of the Pre-hospital Trauma Triage Research Collaborative (PTTRC)

**Affiliations:** 1 Department of Surgery, University Medical Center Utrecht, Utrecht, The Netherlands; 2 Department of Surgery, Maastricht University Medical Center, Maastricht, The Netherlands; 3 Network Acute Care Limburg, Maastricht University Medical Center, Maastricht, The Netherlands; 4 Center for Artificial Intelligence in Medicine & Imaging, Stanford University, Stanford, USA; 5 Department of Surgery, Erasmus University Medical Center, Rotterdam, The Netherlands; 6 Department of Surgery, Diakonessenhuis, Zeist/Doorn, Utrecht, The Netherlands

**Keywords:** Inter-hospital transfer, Trauma, Mortality, Emergency Medical Services

## Abstract

**Purpose:**

The importance of treating severely injured patients in higher-level trauma centers is undisputable. However, it is uncertain whether severely injured patients that were initially transported to a lower-level trauma center (i.e., undertriage) benefit from being transferred to a higher-level trauma center.

**Methods:**

This observational study included all severely injured patients (i.e., Injury Severity Score ≥ 16) that were initially transported to a lower-level trauma center within eight ambulance regions. The exposure of interest was whether a patient was transferred to a higher-level trauma center. Primary outcomes were 24-h and 30-day mortality. Generalized linear models including inverse probability weights for several potential confounders were constructed to evaluate the association between transfer status and mortality.

**Results:**

We included 165,404 trauma patients that were transported with high priority to a trauma center, of which 3932 patients were severely injured. 1065 (27.1%) patients were transported to a lower-level trauma center of which 322 (30.2%) were transferred to a higher-level trauma center. Transferring undertriaged patients to a higher-level trauma center was significantly associated with reduced 24-h (relative risk [RR] 0.26, 95%-CI 0.10–0.68) and 30-day mortality (RR 0.65, 0.46–0.92). Similar results were observed in patients with critical injuries (24-h: RR 0.35, 0.16–0.77; 30-day: RR 0.55, 0.37–0.80) and patients with traumatic brain injury (24-h: RR 0.31, 0.11–0.83; 30-day: RR 0.66, 0.46–0.96).

**Conclusions:**

A minority of the undertriaged patients are transferred to a higher-level trauma center. An inter-hospital transfer appears to be safe and may improve the survival of severely injured patients initially transported to a lower-level trauma center.

**Supplementary Information:**

The online version contains supplementary material available at 10.1007/s00068-022-02087-7.

## Introduction

Regionalized trauma systems were established to optimize outcomes of trauma patients. Prior research has demonstrated that direct transportation of severely injured patients from the scene of injury to lower-level trauma centers (i.e., undertriage) is potentially harmful as it is associated with increased mortality and morbidity rates [1–4]. In light of this, the American College of Surgeons Committee on Trauma (ACSCOT) has recommended that trauma systems and Emergency Medical Services (EMS) should strive to achieve an undertriage rate of < 5% [5]. The Dutch Healthcare Institute dictates an undertriage of less than 10%. Field triage is crucial, as the initial destination of trauma patients is generally determined on-scene by EMS professionals. Triage protocols were developed to aid EMS professionals in their decision-making to improve field triage. Despite this effort, many patients are erroneously transported to a lower-level trauma center as most trauma centers worldwide are unable to attain undertriage rates < 5% [6]. An inter-hospital transfer to a higher-level trauma center may be beneficial for severely injured patients that were initially transported to a lower-level trauma center.

Numerous studies have investigated the effect of direct versus indirect presentation at higher-level trauma centers but remain inconclusive on this matter [7, 8]. However, only few studies have investigated the impact of inter-hospital transfers in severely injured undertriaged patients. Two studies reported a survival benefit of inter-hospital transfers [9, 10]. Similar results were found in patients with gunshot wounds [11]. These studies, however, did not exclusively include severely injured patients based on an anatomical reference standard and did not investigate the impact in specific subgroups (i.e., traumatic brain injury).

The objective of the current study was to evaluate the mortality in secondarily transferred severely injured patients in comparison to undertriaged patients that received definitive care in a lower-level trauma center. Additionally, we also evaluated the impact of an inter-hospital transfer in patients with critical injuries, traumatic brain injury, and severe thoracic injury.

## Methods

### Study design and setting

The current study was conducted according to the Strengthening the Reporting of Observational Studies in Epidemiology guidelines [12]. The Medical Ethical Committee of the University Medical Center Utrecht concluded that the Medical Research Involving Human Subjects Act did not apply. Eight EMS regions (Amsterdam-Amstelland, Brabant Midden-West, Brabant-Noord, Gelderland-Zuid, Rotterdam-Rijnmond, Utrecht, Zaanstreek-Waterland, and Zuid-Holland Zuid) and seven trauma regions participated in this study. The EMS regions currently use the 8^th^ version of the National Protocol of Ambulance Services (in Dutch: *‘Landelijk Protocol Ambulancezorg’*), originating from the Field Triage Decision Scheme developed by the ACSCOT [5, 13]. The ground ambulances are staffed by a dedicated driver that is capable to provide medical assistance and a nurse that is licensed to provide advanced life support care. Level-I trauma centers (i.e., higher-level trauma centers) in the Netherlands are capable to treat patients in need of specialized care and meet the criteria as outlined by the ACSCOT [14]. Only these centers are capable to provide 24/7 neurosurgical and cardiothoracic care. Lower-level trauma centers (i.e., level-II and level-III trauma centers) are designated to treat mildly and moderately injured patients. There are seven higher-level trauma centers and 60 lower-level trauma centers in the participating trauma regions. No trauma patients are transported to or treated at non-trauma centers in the Netherlands.

### Selection of participants

**Fig. 1:**
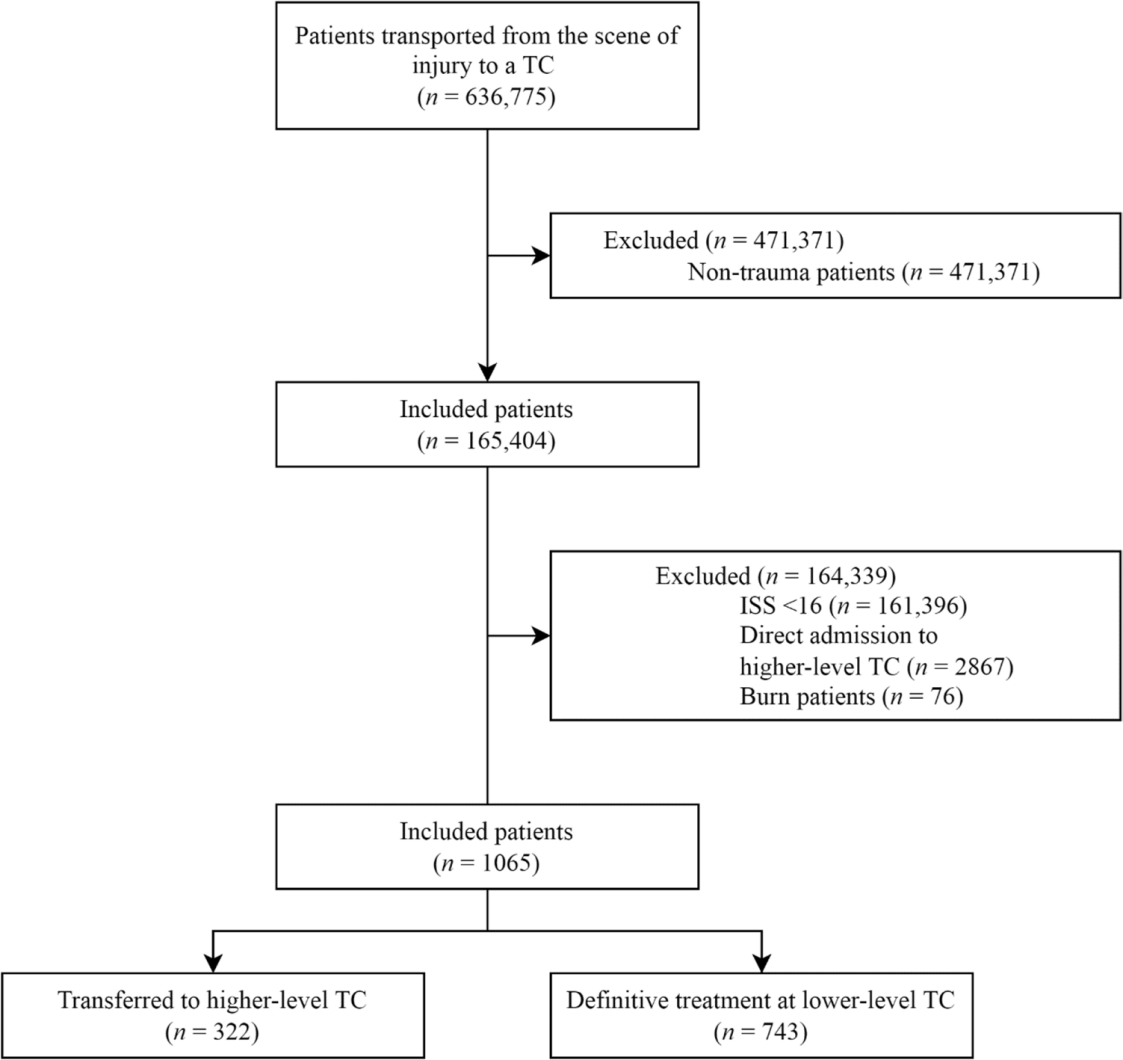
Patient inclusion strategy. *EMS* Emergency Medical Services, *TC* Trauma Center, *ISS* Injury Severity Score

Patients transported in the participating EMS regions between Jan 2015 and Dec 2017 were included. Non-urgent cases (i.e., scheduled transports) were not included as we assume that these patients are not severely injured. Non-trauma patients were excluded. Burn patients are generally transported to the nearest trauma center [13, 15]. We excluded these patients because of different triage strategies. Severely injured patients that were initially transported to a lower-level trauma center remained eligible for inclusion (Fig. 1). A previously developed selection tool was used to identify trauma patients in unfiltered EMS records with an accuracy of 98.9% [16]. Free text fields filled out by EMS professionals were analyzed with a recurrent neural network. These results were incorporated in a prediction model that also includes several variables, such as mechanism of injury and chief complaint.

### Outcomes and definitions

Main outcomes were 24-h and 30-day mortality. The exposure of interest was transfer status. The Injury Severity Score (ISS) was based on the Abbreviated Injury Scale (AIS) version 2005 (update 2008) [17]. A severely injured patients was defined as an ISS of equal to or greater than 16. Subgroup analyses were conducted in critically injured patients (ISS ≥ 25), patients with traumatic brain injury (AIS score ≥ 3 in head region), and patients with severe thoracic injury (AIS score ≥ 3 in thoracic ISS region).

### Data collection

Electronic EMS records contained patient demographics, dispatch priority, and transport destination. In the Netherlands, the following two levels of urgent dispatch priority exist: highest priority (i.e., lights and sirens) and high priority (i.e., urgent transport without lights or sirens). Trained data registrars at the trauma regions record data for all admitted trauma patients including vital parameters measured in the emergency department, mechanism of injury, AISs, ISSs, and mortality status. Mechanism of injury included high-energetic falls (i.e., ≥ 2–3 times the patient’s length) and motorized vehicle crashes. EMS and in-hospital records were linked using a unique EMS identifier. For patients with a missing identifier, a prediction model, developed in prior research, was used [16]. Zip codes of hospitals were converted into latitude and longitude coordinates. The haversine formula was used to select the two nearest higher-level trauma centers for every patient. Driving distances to these trauma centers were computed with Bing Maps (Microsoft^™^, Redmond, US), accounting for day of the week and hour of the day [18]. The smallest value was chosen as the driving distance to the nearest higher-level trauma center. Zip-code based estimates have been demonstrated to be an adequate estimate of the actual driving distance [19].

### Statistical analyses

Statistical analyses were conducted in R statistical software (R version 4.0.3) [20]. Variables with missing values included respiratory rate (31.1%), systolic blood pressure (29.9%), Glasgow Coma Scale score (17.8%), and dispatch priority (9.4%). Missing data were multiply imputed, using the R-package *micemd* [21]. We generated 48 imputed datasets based on 20 iterations per imputation. The predictor matrix that was developed to impute the missing values included, among others, patient demographics, mechanism of injury, pre-hospital (e.g., systolic blood pressure measured on-scene) and hospital vital parameters (e.g., Glasgow Coma scale in the emergency department), injuries (e.g., head or thoracic injury), ISS, and mortality. Descriptive statistics were depicted in median and interquartile ranges (IQR) for continuous variables, and proportions for categorical variables. Entropy balancing was conducted with the *weightit* R-package [22–24]. Entropy balancing has proved to be a robust method to enforce covariate balancing. Weights were computed using the variables age, gender, dispatch priority, vital parameters measured in the emergency department, mechanism of injury, ISS, severe injuries (i.e., AIS score ≥ 3 per ISS region), and driving distance to the nearest higher-level trauma center. We hypothesize that these prognostic factors are potentially associated with the decision whether a patient is transferred to a higher-level trauma center and patient outcomes. Restricted cubic splines were used to account for non-linearity. Weights were truncated to the 99th percentile, to account for extreme weights. Truncation of weights potentially introduces bias but greatly improves precision [25]. Balance of the covariates with regard to transfer status was assessed with the *cobalt* R-package [22]. Covariates with a standardized mean difference of < 0.1 were considered adequately balanced. A generalized linear model was constructed including the aforementioned weights based on the prognostic factors. The analyses were performed on each of the multiply imputed datasets. We calculated point estimates by pooling the results of these analyses. We argue that patients who died in the emergency department had no reasonable chance of being transferred. Therefore, 24-h and 30-day mortality excluding death in the emergency department served as a sensitivity analysis (Table 1).

**Table 1:** Cohort characteristics

	Transfer group (*n* = 322)	Non-transfer group (*n* = 743)
Age, years	57.9 (39.5–70.6)	69.5 (52.1–82.7)
Age < 16 years	23 (7.1)	5 (0.7)
Age ≥ 65 years	114 (35.4)	430 (57.9)
Female gender	109 (33.9)	327 (44.0)
Highest dispatch priority	252 (78.3)	483 (65.0)
Vital signs		
Systolic blood pressure, mmHg	140 (122–160)	141 (125–162)
Systolic blood pressure < 90 mmHg	8 (2.5)	18 (2.4)
Respiratory rate, breaths per min	16 (14–20)	16 (14–19)
Respiratory rate > 29 or < 10 breaths per min	6 (1.9)	25 (3.4)
Glasgow Coma Scale score	15 (13–15)	15 (14–15)
Glasgow Coma Scale score < 14	128 (39.8)	149 (20.1)
Mechanism of injury		
High-energetic fall	64 (19.8)	92 (12.4)
Motorized vehicle crash	22 (6.8)	51 (6.9)
Penetrating injury	2 (0.1)	3 (0.0)
ISS	21 (17–26)	18 (17–22)
ISS ≥ 25	143 (44.4)	163 (21.9)
Traumatic brain injury	233 (72.4)	398 (53.6)
Severe injury (AIS score ≥ 3) per ISS region		
Head and/or neck	258 (80.1)	440 (59.2)
Thorax	65 (20.2)	277 (37.3)
Abdomen	25 (7.8)	73 (9.8)
Extremities	34 (10.6)	145 (19.5)

*ISS* Injury Severity Score, *AIS* Abbreviated Injury Scale, *TC* Trauma Center, *min* minute.

Data are median (IQR) or *n* (%).Variables with missing data were systolic blood pressure (30.9%), respiratory rate (30.7%), and Glasgow Coma Scale score (18.5%).

## Results

### Baseline characteristics

During the study period, 636,775 patients were transported from the scene of injury to a trauma center (Fig. 1). After excluding non-trauma patients, mildly and moderately injured (i.e., ISS < 16) patients, patients directly admitted to a higher-level trauma center, and burn patients, 1065 severely patients that were initially transported to a lower-level trauma center remained. Of these patients, 322 (30.2%) were transferred to a higher-level trauma center. Transferred patients were more often children (7.1 vs. 0.7%) and less frequent elderly (35.4 vs. 57.9%) compared to patients that received definitive care in a lower-level trauma center. Also, transferred patients more frequent had an impaired Glasgow Coma Scale (39.8 vs. 20.1%), traumatic brain injury (72.4 vs. 53.6%), and critical injuries (44.4% vs. 21.9%). These patients had fewer severe injuries to the thorax (20.2 vs. 37.3%) and extremities (10.6% vs. 19.5%). The median ISS was 21 (IQR, 17–26) in the transfer group and 18 (17–22) in the non-transfer group.

### Outcomes

Median driving distance from the initial destination hospital to the nearest higher-level trauma center was 25.3 km (IQR, 11.0–52.8) in the transfer group and 25.0 km (8.7–38.6) in the non-transfer group (Table 2). Median time to arrival in definitive trauma center was 249.8 min (IQR, 192.9–380.1) in the transfer group and 41.5 min (IQR, 34.1–50.7) in the non-transfer group. Forty (12.4%) patients were transferred to a higher-level trauma center for a craniotomy. 24-h mortality rates were 3.1% in the transfer group and 3.6% in the non-transfer group, whereas 30-day mortality rates for both groups were 12.1% and 15.5%, respectively. Six patients died in the non-transfer group, whilst none of the transferred patients deceased. Covariates were adequately balanced (i.e., standardized mean difference < 0.1) using inverse probability weighting (Supplementary Table 1). The generalized linear weighted model is demonstrated in Table 3. A transfer to a higher-level trauma center was significantly associated with reduced 24-h mortality (relative risk [RR] 0.26, 95% CI, 0.10–0.68) and 30-day mortality (RR 0.65, 0.46–0.92).

**Table 2:** Outcomes of transferred and non-transferred patients

	Transfer group (*n* = 322)	Non-transfer group (*n* = 743)
Driving distance to nearest higher-level TC, km	25.3 (11.0–52.8)	25.0 (8.7–38.6)
Time to arrival at TC*, min	249.8 (192.9–380.1)	41.5 (34.1–50.7)
Emergency intervention†		
Damage control laparotomy	3 (0.9)	1 (0.1)
Extraperitoneal pelvic packing	1 (0.3)	1 (0.1)
Radiological intervention	4 (1.2)	6 (0.8)
Craniotomy	40 (12.4)	0 (0)
Coniotomy/cricothyrotomy	1 (0.3)	0 (0)
Hospital length of stay, days		
Lower-level TC	0.2 (0.1–0.5)	6.0 (2.2–10.4)
Higher-level TC	6.1 (2.9–13.2)	N/A
ICU admission		
Lower-level TC	6 (1.9)	173 (23.3)
Higher-level TC	180 (55.9)	N/A
24 h mortality	10 (3.1)	27 (3.6)
24 h mortality (excluding death in ED)	10 (3.1)	21 (2.8)
30 day mortality	39 (12.1)	115 (15.5)
30-day mortality (excluding death in ED)	39 (12.1)	109 (14.7)

*ISS* Injury Severity Score, *AIS* Abbreviated Injury Scale, *TC* Trauma Center, *min* minute, *N/A* Not applicable, *ED* Emergency Department, *ICU* Intensive Care Unit.

Data are median (IQR) or *n* (%).

* Time to arrival at TC was missing in 12.6% of the patients.

† Emergency interventions in the transfer group solely include interventions performed at the definitive (i.e., higher-level) trauma center.

**Table 3:** Generalized linear model including inverse probability weights

Variable	All patients	ISS ≥ 25	Traumatic brain injury	Severe thoracic injury
	Relative risk (95% CI)	Relative risk (95% CI)	Relative risk (95% CI)	Relative risk (95% CI)
24 h mortality				
Non-transfer	Reference	Reference	Reference	Reference
Transfer to higher-level TC	0.26 (0.10–0.68)*	0.35 (0.16–0.77)*	0.31 (0.11–0.83)*	N/A†
24 h mortality (excluding death in ED)				
Non-transfer	Reference	Reference	Reference	Reference
Transfer to higher-level TC	0.30 (0.11–0.82)*	0.40 (0.18–0.89)*	0.38 (0.14–1.05)	N/A†
30-day mortality				
Non-transfer	Reference	Reference	Reference	Reference
Transfer to higher-level TC	0.65 (0.46–0.92)*	0.55 (0.37–0.80)*	0.66 (0.46–0.96)*	0.61 (0.25–1.47)
30-day mortality (excluding death in ED)				
Non-transfer	Reference	Reference	Reference	Reference
Transfer to higher-level TC	0.67 (0.47–0.96)*	0.56 (0.38–0.83)*	0.65 (0.45–0.95)*	0.70 (0.29–1.67)

*ISS* Injury Severity Score, *TC* Trauma Center, *ED* Emergency Department.

* Significantly associated.

† Not available due to too few cases.

### Sensitivity analyses

After exclusion of patients that died in the emergency department, transfer status remained significantly associated with 24-h (RR 0.30, 95%-CI, 0.11–0.82) and 30-day mortality (RR 0.67, 0.47–0.96). Inter-hospital transfer was also associated with reduced mortality in patients with critical injuries (24-h: RR 0.35, 0.16–0.77; 30-day: RR 0.55, 0.37–0.80) and patients with a traumatic brain injury (24-h: RR 0.31, 0.11–0.83; 30-day: RR 0.66, 0.46–0.96). Transfer status was not associated with mortality in patients with severe thoracic injury.

## Discussion

In this prospective cohort study, we investigated the effect of inter-hospital transfers in severely injured patients that were initially transported to a lower-level trauma center. We demonstrated that transferring undertriaged patients to a higher-level trauma center is significantly associated with reduced mortality. Moreover, we observed that this association was also present in patients with traumatic brain injury or critical injuries. Only 30.2% of the undertriaged patients were transferred to a higher-level trauma center. The driving distances to the nearest higher-level trauma center of transferred patients were comparable to those that received definitive treatment in a lower-level trauma center. We considered that patients who died in the emergency department of the initial destination had no reasonable chance of being transferred. Therefore, we also evaluated the effect of inter-hospital transfers on mortality excluding the patients that died in the emergency department of lower-level trauma centers. Transferring severely injured patients to a higher-level trauma center remained significantly associated with reduced 24-h and 30-day mortality. This was also the case in patients with critical injuries.

Solely higher-level (i.e., level-I) trauma centers in the Netherlands are capable to provide neurosurgical and cardiothoracic care. It therefore, seems evident that patients with a traumatic brain injury benefit from an inter-hospital transfer to a higher-level trauma center, which was endorsed by our results. However, only 37% of these patients are transported to a higher-level trauma center. We did not observe a favorable effect of transferring patients with severe thoracic injuries. Our findings, therefore, suggest that an inter-hospital transfer is mainly beneficial for patients with critical injuries or a traumatic brain injury.

The broad inclusion criteria are a major strength of this study. We included all trauma patients transported in eight different EMS regions. These patients were linked to in-hospital records from trauma centers within seven participating regions. All trauma centers contributed to the prospective and standardized data collection and all patients admitted to these centers were included in the dataset. Only 0.2% of the patients were transported to a non-participating trauma region, reducing the chance of selection bias. Moreover, we have verified in data from prior research that patients that were discharged from the emergency department were not severely injured [26]. The participating EMS regions cover urban, suburban and rural areas, thereby contributing to the generalizability of our results. Also, the combination of the many explanatory variables in our dataset and the robust balancing methods enabled us to ascertain the causal relationship between inter-hospital transfers and mortality [23].

It has been suggested that defining a severely injured patient based on resource use could be a superior alternative to the ISS [27, 28]. We hypothesized that it would be invalid to use a resource-based reference standard in the current study as some resources are unavailable in lower-level trauma centers. Also, the lower volume of severely injured patients in lower-level trauma centers affects the use of and experience with certain resources. Furthermore, burn patients were excluded because these patients are generally transported to the nearest trauma center and specialized burn centers in the Netherlands are not considered higher-level trauma centers [15].

It has been reported that severely injured patients benefit from treatment in a higher-level trauma center [1–4]. In light of this, two systematic reviews, including over 40 studies, have compared direct versus indirect presentation at a higher-level trauma center to demonstrate the importance of inter-hospital transfers, but remained inconclusive on this matter [7, 8]. Our study differs from these prior investigations because we compared severely injured patients that were transferred to a higher-level trauma center with patients that received definitive treatment in a lower-level trauma center. We aimed to endorse the importance of inter-hospital transfers, rather than questioning whether undertriage is harmful.

There is little evidence on the impact of inter-hospital transfers among patients that were initially transported to a lower-level trauma center. Our findings are in strong agreement with two studies that investigated the impact of inter-hospital transfers in severely injured patients [9, 10]. Newgard and colleagues (2007) demonstrated that an inter-hospital transfer from a nontertiary center to a higher-level (i.e., level I or level II in the US) trauma center is associated with reduced in-hospital mortality (odds ratio 0.67, 95%-CI, 0.48–0.94). Garwe et al. (2010) demonstrated similar results (hazard ratio 0.38, 0.30–0.50) [9]. These investigations did not exclusively investigate severely injured patients, as moderately injured patients [9] or patients with hypotension (i.e., systolic blood pressure < 90 mmHg) or certain comorbid conditions were also included in these studies [10]. We solely included severely injured patients defined by an ISS ≥ 16, as recommended by the ACSCOT [5]. We hypothesized that inclusion based on this anatomical reference standard is a better alternative as there is a large body of evidence that demonstrates the importance of treatment of these patients in a higher-level trauma center [1–4]. Also, we observed that patients that were transferred were younger and more often males, which suggests that patient demographics are incorporated into the transfer decision-making. Prior research reported similar findings [9, 10]. Additionally, it has been suggested that the first hours after trauma are critical in severely injured patients [29–31]. Therefore, we also evaluated the impact of inter-hospital transfers on 24-h mortality. Furthermore, transferred patients arrived approximately 3.5 h later at the definitive trauma center. We hypothesize that the benefit of an inter-hospital transfer outweighs the additional time to definitive care. Our results indicate that an inter-hospital transfer is safe and beneficial for severely injured patients. These findings could aid physicians in the emergency department of lower-level trauma centers in their decision whether to transfer a patient or not.

Our study has the following limitations: First, some of the covariates had a substantial amount of missing data (e.g., systolic blood pressure). We used multiple imputation to account for missing data. The predictor matrix that was used for imputation included, among others, pre-hospital measured vital signs, generating a representational dataset. Time to arrival at the definitive trauma center was unavailable for 12.6% of the patients, because they were transported by EMS professionals from non-participating regions. We did not impute these missing values as it could be affected by many unmeasured factors (e.g., traffic congestion, crowding at the emergency department). However, we assume that the complete cases (87.4%) provide an adequate estimate of the time needed for an inter-hospital transfer. Also, as the time to arrival at the definitive trauma center was not incorporated in the models, we assume that our results were not affected by these missing values. Second, all ground ambulances in the Netherlands are staffed by licensed nurses, which limits the generalizability of our results to regions with a physician-staffed EMS. Third, due to a limited sample size, we were unable to determine the association of transfer status and 24-h mortality in patients with severe thoracic injuries. Fourth, it is difficult to fully account for all factors explaining the prognostic differences between patients that were transferred to a higher-level trauma center and patients that received definitive care in a lower-level trauma center. Patients with the worst prognosis and poor outcomes require more specialized care and are, therefore, more likely to be transferred to a higher-level trauma center. Conversely, it is possible that mostly trauma patients that were stable enough after initial care at the lower-level trauma center (i.e., patients with improved outcomes) were transferred. The decision whether a patient is transferred or not could have been affected by prognostic factors that were unavailable. Although our generalized linear models included weights for many confounding variables, unmeasured confounding is a common limitation in observational research that these models, similar to conventional regression, could not account for.

## Conclusions

A minority of the severely injured patients that were initially transported to a lower-level trauma center are transferred to a higher-level trauma center. An inter-hospital transfer appears to be safe and may even improve the survival of severely injured patients initially transported to a lower-level trauma center. Our findings suggest that mainly critically injured patients and patients with a traumatic brain injury benefit from an inter-hospital transfer to a higher-level trauma center.

## Supplementary Information

Below is the link to the electronic supplementary material.

Supplementary file1 (DOCX 17 KB)

## Data Availability

Available upon reasonable request, approval of the participating EMS and trauma regions, and provided that appropriate ethical approval is sought and approved.

## Code availability

Available upon request.

## References

[1] MacKenzie EJ, Rivara FP, Jurkovich GJ, Nathens AB, Frey KP, Egleston BL (2006). A national evaluation of the effect of trauma-center care on mortality. N Engl J Med.

[2] Staudenmayer K, Weiser TG, Maggio PM, Spain DA, Hsia RY (2016). Trauma center care is associated with reduced readmissions after injury. J Trauma Acute Care Surg.

[3] Polites SF, Leonard JM, Glasgow AE, Zielinski MD, Jenkins DH, Habermann EB (2018). Undertriage after severe injury among United States trauma centers and the impact on mortality. Am J Surg.

[4] Cudnik MT, Newgard CD, Sayre MR, Steinberg SM (2009). Level I versus level II trauma centers: an outcomes-based assessment. J Trauma-Injury Infect Crit Care.

[5] American College of Surgeon Committee on Trauma. Resources for Optimal Care of the Injured Patient. Chicago. 2014. https://www.facs.org/media/yu0laoqz/resources-for-optimal-care.pdf. Accessed 19 Mar 2022.

[6] van Rein EAJ, van der Sluijs R, Houwert RM, Gunning AC, Lichtveld RA, Leenen LPH (2018). Effectiveness of prehospital trauma triage systems in selecting severely injured patients: Is comparative analysis possible?. Am J Emerg Med.

[7] Pickering A, Cooper K, Harnan S, Sutton A, Mason S, Nicholl J (2015). Impact of prehospital transfer strategies in major trauma and head injury: systematic review, meta-analysis, and recommendations for study design. J Trauma Acute Care Surg.

[8] Hill AD, Fowler RA, Nathens AB (2011). Impact of interhospital transfer on outcomes for trauma patients: a systematic review. J Trauma.

[9] Garwe T, Cowan LD, Neas B, Cathey T, Danford BC, Greenawalt P (2010). Survival benefit of transfer to tertiary trauma centers for major trauma patients initially presenting to nontertiary trauma centers. Acad Emerg Med.

[10] Newgard CD, McConnell KJ, Hedges JR, Mullins RJ (2007). The benefit of higher level of care transfer of injured patients from nontertiary hospital emergency departments. J Trauma.

[11] Renson A, Schubert FD, Gabbe LJ, Bjurlin MA (2019). Interfacility transfer is associated with lower mortality in undertriaged gunshot wound patients. J Surg Res.

[12] von Elm E, Altman DG, Egger M, Pocock SJ, Gøtzsche PC, Vandenbroucke JP (2007). The Strengthening the Reporting of Observational Studies in Epidemiology (STROBE) statement: guidelines for reporting observational studies. Lancet.

[13] Ambulancezorg Nederland. Landelijk Protocol Ambulancezorg (versie 8.1). Zwolle 2016. https://www.ambulancezorg.nl/themas/kwaliteit-van-zorg/protocollen-en-richtlijnen/landelijk-protocol-ambulancezorg. Accessed 21 Mar 2022.

[14] Lansink KW, Gunning AC, Spijkers AT, Leenen LP (2013). Evaluation of trauma care in a mature level I trauma center in the Netherlands: outcomes in a Dutch mature level I trauma center. World J Surg.

[15] Dutch Burns Foundation. Guideline First Aid of Burn Patients in the Acute Phase (first 24 hours) of Burn Accidents and Referral to a Burn Center. 2020. https://brandwondenzorg.nl/wp-content/uploads/2020/10/Herziening-richtlijn-Eerste-opvang-van-brandwondpati%C3%ABnten_DEF.pdf. Accessed 21 Mar 2022.

[16] van der Sluijs R, Lokerman RD, Waalwijk JF, de Jongh MAC, Edwards MJR, den Hartog D (2020). Accuracy of pre-hospital trauma triage and field triage decision rules in children (P2–T2 study): an observational study. Lancet Child Adolesc Health..

[17] Gennarelli TA, Wodzin E. Abbreviated Injury Scale 2005: update 2008 Des Plaines, IL: Association for the Advancement of Automative Medicine; 2008.

[18] Bing Maps. Microsoft Cooperation, Redmond, USA. 2021. https://www.bing.com/maps. Accessed 21 Mar 2022.

[19] Jarman MP, Sturgeon D, Mathews I, Uribe-Leitz T, Haider AH (2019). Validation of zip code-based estimates of ambulance driving distance to control for access to care in emergency surgery research. JAMA Surg.

[20] R Development Core Team. R: A Language and Environment for Statistical Computing. Vienna, Austria: R Foundation for Statistical Computing; 2021.

[21] Audigier V, Resche-Rigon M. Micemd: Multiple Imputation by Chained Equations with Multilevel Data. Version 1.6.0. 2019. https://rdrr.io/cran/micemd. Accessed 15 Apr 2022.

[22] Greifer N. Weightit: Weighting for Covariate Balance in Observational Studies. Version 0.10.2. 2020. https://rdrr.io/cran/WeightIt. Accessed 15 Apr 2022.

[23] Zhao QY, Percival D (2017). Entropy Balancing is Doubly Robust. J Causal Inference..

[24] Hainmueller J (2017). Entropy balancing for causal effects: a multivariate reweighting method to produce balanced samples in observational studies. Polit Anal.

[25] Cole SR, Hernan MA (2008). Constructing inverse probability weights for marginal structural models. Am J Epidemiol.

[26] Voskens FJ, van Rein EAJ, van der Sluijs R, Houwert RM, Lichtveld RA, Verleisdonk EJ (2018). Accuracy of prehospital triage in selecting severely injured trauma patients. JAMA Surg.

[27] Lerner EB, Willenbring BD, Pirrallo RG, Brasel KJ, Cady CE, Colella MR (2014). A consensus-based criterion standard for trauma center need. J Trauma Acute Care Surg.

[28] Newgard CD, Hedges JR, Diggs B, Mullins RJ (2008). Establishing the need for trauma center care: anatomic injury or resource use?. Prehosp Emerg Care.

[29] Lefering R, Paffrath T, Bouamra O, Coats TJ, Woodford M, Jenks T (2012). Epidemiology of in-hospital trauma deaths. Eur J Trauma Emerg Surg.

[30] Lansink KW, Gunning AC, Leenen LP (2013). Cause of death and time of death distribution of trauma patients in a Level I trauma centre in the Netherlands. Eur J Trauma Emerg Surg.

[31] Demetriades D, Kimbrell B, Salim A, Velmahos G, Rhee P, Preston C (2005). Trauma deaths in a mature urban trauma system: is "trimodal" distribution a valid concept?. J Am Coll Surg.

