# Supplementary material for: The influence of inter-hospital transfers on mortality in severely injured patients
Source: Eur J Trauma Emerg Surg. 2022 Sep 1;49(1):441–9. doi: 10.1007/s00068-022-02087-7 (PMC9925487; doi:10.1007/s00068-022-02087-7)
Supplement: Supplementary file 1 — Supplementary file1 (DOCX 17 KB) [file 68_2022_2087_MOESM1_ESM.docx]

**Supplementary content**Supplement to: Waalwijk JF, Lokerman RD, van der Sluijs R, et al. Evaluating the effect of inter-hospital transfers on mortality in severely injured patients. **Supplementary Table 1.** Standard mean difference before and after weighting.

| **Supplementary Table 1.**  **Standard mean difference before and after weighting.** | | | | | |  | |  | |  | |
| --- | --- | --- | --- | --- | --- | --- | --- | --- | --- | --- | --- |
|  | **All patients** | | **ISS ≥25** | | **Traumatic brain injury** | | | | **Severe thoracic injury** | | |
|  | **Unadjusted correlation** | **Adjusted correlation** | **Unadjusted correlation** | **Adjusted correlation** | **Unadjusted correlation** | | **Adjusted correlation** | | **Unadjusted correlation** | | **Adjusted correlation** |
|  |  |  |  |  |  | |  | |  | |  |
| Age | -0.5232 | -0.0381 | -0.4835 | -0.0045 | -0.7497 | | -0.0295 | | -0.3853 | | -0.1139 |
| Age* | -0.5683 | -0.0534 | -0.6182 | -0.0023 | -0.7914 | | -0.0328 | | -0.3604 | | -0.1230 |
| Gender | 0.1016 | -0.0041 | 0.0396 | -0.0019 | 0.1043 | | 0.0039 | | 0.0337 | | -0.0110 |
| Highest dispatch priority | 0.1335 | 0.0046 | 0.2127 | 0.0015 | 0.1867 | | -0.0019 | | 0.1145 | | -0.0124 |
| Vital signs |  |  |  |  |  | |  | |  | |  |
| Systolic blood pressure | -0.0432 | -0.0164 | -0.0024 | 0.0084 | -0.1580 | | -0.0147 | | -0.2737 | | -0.0822 |
| Systolic blood pressure* | -0.0565 | -0.0152 | -0.0952 | 0.0074 | -0.1674 | | -0.0032 | | -0.2061 | | -0.0636 |
| Respiratory rate | 0.0267 | -0.0070 | 0.0213 | 0.0032 | 0.0967 | | 0.0217 | | -0.0771 | | -0.0357 |
| Glasgow Coma Scale | -0.6300 | -0.0104 | -0.5379 | -0.0108 | -0.8212 | | -0.0445 | | -0.4107 | | 0.0469 |
| Mechanism of injury |  |  |  |  |  | |  | |  | |  |
| High energetic fall | 0.0749 | 0.0038 | 0.0680 | 0.0007 | 0.0654 | | 0.0017 | | 0.1282 | | 0.0191 |
| Motorized vehicle crash | -0.0003 | 0.0019 | 0.0287 | 0.0001 | 0.0160 | | -0.0029 | | 0.0740 | | -0.0003 |
| Penetrating injury | 0.0022 | 0.0001 | 0.0079 | 0.0000 | 0.0086 | | 0.0005 | | -0.0037 | | -0.0030 |
| ISS | 0.3643 | 0.0216 | 0.0908 | -0.0002 | 0.4249 | | 0.0352 | | 0.6173 | | 0.0276 |
| Severe injury (AIS score ≥3) per ISS region |  |  |  |  |  | |  | |  | |  |
| Head and neck | 0.2090 | 0.0151 | 0.1799 | 0.0056 | 0.0050 | | 0.0006 | | 0.2081 | | 0.0237 |
| Thorax | -0.1709 | -0.0070 | -0.0733 | -0.0022 | -0.0359 | | -0.0017 | | N/A | | N/A |
| Abdomen | -0.0206 | -0.0005 | -0.0160 | -0.0027 | 0.0071 | | 0.0004 | | 0.0586 | | 0.0405 |
| Extremities | -0.0869 | -0.0039 | -0.0266 | -0.0028 | -0.0522 | | 0.0029 | | -0.0132 | | 0.0108 |
| Driving distance to nearest TC | 0.2599 | -0.0011 | 0.2748 | 0.0019 | 0.2450 | | 0.0172 | | 0.2421 | | 0.0259 |
| ISS: Injury Severity Score; AIS: Abbreviated Injury Scale; TC: Trauma Center; *restricted cubic splines with three knots | | | | | | | | | | | |
|  | | | | | | | |  | |  | |
